# Kinematic real-time feedback is more effective than traditional teaching method in learning ankle joint mobilisation: a randomised controlled trial

**DOI:** 10.1186/s12909-016-0789-8

**Published:** 2016-10-06

**Authors:** Manuel González-Sánchez, Maria Ruiz-Muñoz, Ana Belén Ávila-Bolívar, Antonio I. Cuesta-Vargas

**Affiliations:** 1 Departamento de Fisioterapia, Universidad de Málaga, Instituto de Investigación Biomédica de Málaga (IBIMA), Arquitecto Francisco Peñalosa s/n. (ampliación Campus Teatinos), 29071 Málaga, Spain; 2 Departamento de Enfermería y Podología, Universidad de Málaga, Instituto de Investigación Biomédica de Málaga (IBIMA), Málaga, Spain; 3 University de Malaga, Malaga, Spain; 4 School of Clinical Sciences at Queensland University, Brisbane, Australia

**Keywords:** Inertial sensor, Manual therapy, Education, Training

## Abstract

**Background:**

To analyse the effect of real-time kinematic feedback (KRTF) when learning two ankle joint mobilisation techniques comparing the results with the traditional teaching method.

**Methods:**

Double-blind randomized trial. Settings: Faculty of Health Sciences. Participants: undergraduate students with no experience in manual therapy. Each student practised intensely for 90 min (45 min for each mobilisation) according to the random methodology assigned (G1: traditional method group and G2: KRTF group). G1: an expert professor supervising the student’s practice, the professorstudent ratio was 1:8. G2: placed in front of a station where, while they performed the manoeuvre, they received a KRTF on a laptop. Outcome measures: total time of mobilisation, time to reach maximum amplitude, maximum angular displacement in the three axes, maximum and average velocity to reach the maximum angular displacement, average velocity during the mobilisation.

**Results:**

Among the pre-post intervention measurements, there were significant differences within the two groups for all outcome variables, however, G2 (KRTF) achieved significantly greater improvements in kinematic parameters for the two mobilisations (significant increase in displacement, velocity and significant reduction in the mobilisations runtime) than G1. Ankle plantar flexion: G1′s measurement stability (post-intervention) ranged between 0.491 and 0.687, while G2′s measurement stability ranged between 0.899 and 0.984. Ankle dorsal flexion mobilisation: G1 the measurement stability (post-intervention) ranged from 0.543 and 0.684 while G2 ranged between 0.899 and 0.974.

**Conclusion:**

KRTF was proven to be more effective tool than traditional teaching method in the teaching - learning process of two joint mobilisation techniques.

**Trial registration:**

NCT02504710.

## Background

During the process of learning and acquiring motor skills, it is normal that a student makes mistakes [1]. The identification of these errors and their correction is essential in order that the student remains engaged in the training of this ability and a transfer of learning takes place [1, 2]. In the process of the acquisition of skills four different stages, which are interconnected, are generated, namely learning, error detection, error correction and training [2]. The traditional teaching method in which the professor gives a demonstration and the student repeats what he or she observes has some limitations: the identification of errors and subsequent corrections that the student receives depends on the professor-student ratio, and the information received by the student is always subjective [2, 3]. To overcome these limitations, new teaching-learning strategies to acquire new abilities/skills are being developed based on the use of instruments that provide feedback in real time with objective information, according to specific and useful parameters of the ability/skill [4–6]. These new strategies could be used by all health professionals because they allow the student to identify errors in the implementation and to correct them, increasing their competence and autonomy during the learning process [6].

Previous studies have used different tools/instruments to provide real-time feedback, such as instrumented treatment tables [6–13], handheld force transducers [6–14], instrumented manikins [8, 15] and inertial sensors (IS) [5]. IS offer three-dimensional kinematic variables and could provide very good real-time feedback to teach/learn manual skills that have to be performed accurately and swiftly [5, 16–19]. An example of these kinds of skills could be manual therapy techniques on peripheral joints.

The aim of this study was to analyse the effect of kinematic real-time feedback (KRTF) when learning two ankle joint mobilization techniques (dorsiflexion and plantar flexion with talus displacement towards anterior and posterior, respectively), comparing the results with those of a traditional teaching method, such as mobilization. The hypothesis is that KRTF promotes greater development in the learning of the two ankle joint mobilization techniques analysed in this study compared with the traditional teaching method.

## Methods

### Design

A double-blind randomized trial compared the effect of KRTF with the traditional teaching method when learning two ankle joint mobilization techniques (dorsiflexion and plantar flexion with displacement of the talus). Data collection of the study was conducted between 15 September 2015 and 30 November 2015 at a Faculty of Health Sciences.

### Participants

**Fig. 1:**
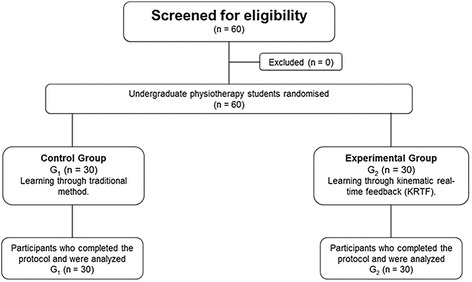
Design and flow of participants through the trial

The inclusion criteria to select participants was undergraduate students with no experience in manual therapy. Having some experience in manual therapy, even using different techniques than those chosen in this study, was the only exclusion criterion. A software that generates random numbers was used and students were randomized into two distinct groups, with one group to use the traditional teaching method (G1: control group), and the other to use KRTF (G2: experimental group) (Fig. 1). The participants’ assignment in each group was conducted by a blinded investigator.

### Ethical approval statement

Before starting the protocol, participants gave informed consent and were informed that their rights were protected. The study was conducted according to the ethical principles of the Helsinki Declaration (Ethical Principles for Medical Research Involving Human Subjects) and was approved by he Ethical Committee of the University of Malaga.

### Intervention

For kinematic registration of the selected mobilizations, two IS (Inertial Cube 3 (Intersense Inc., USA)) with a sampling frequency of 180 Hz were used. One sensor was placed on the back of the heel, while the other was placed on the posterior-distal third of the leg. Sensors were placed such that the origin of coordinates (X, Y, Z) (0, 0, 0) was placed in the left posterior-inferior vertex.

The mobilization techniques examined were ankle dorsiflexion and plantar flexion with talus displacement towards the anterior and posterior, respectively. To this end, the patient was placed in a prone position with knee flexed at 45°–60°. The manual therapist placed the patient’s sole of the foot to his or her ribs, resulting in a closed kinetic chain. Then, he or she placed their first hand angled closely to the front side of the talar neck with the other hand placed at the same location on the back side (thumb and forefingers of each hand should be placed below the malleolus). From a neutral ankle position, the manual therapist initiated ankle dorsiflexion and plantar flexion through body rotations. In the middle range of motion, a talus slip was caused. During ankle plantar flexion, the hand placed on the front side of the talar neck performed a traction, while the hand placed on the backside performed a bone push. Also during ankle dorsiflexion, the hand located on the back face of the talar neck performed traction while the hand placed on the talus pushed.

**Fig. 2:**
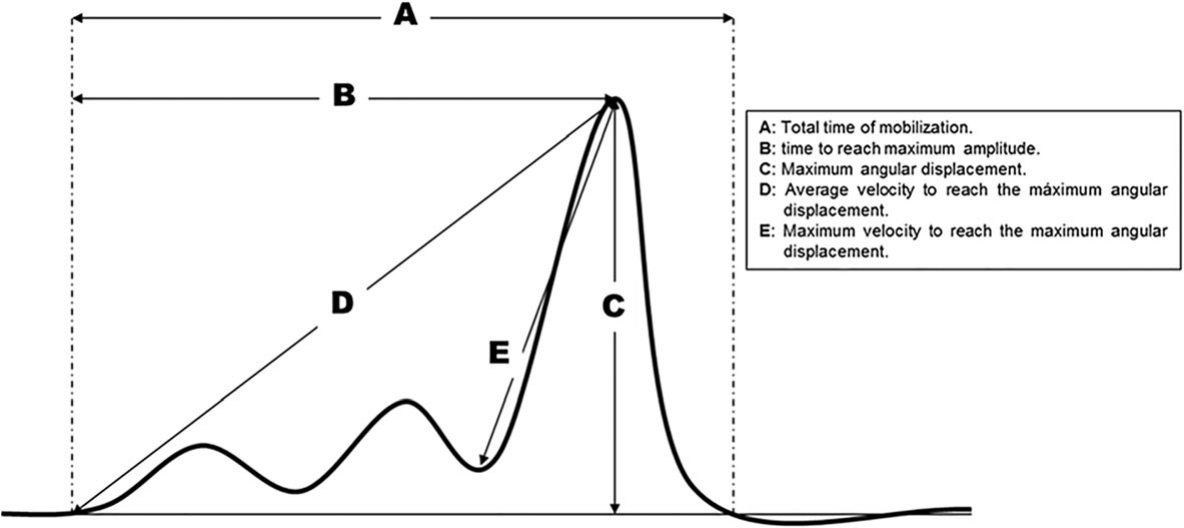
Stylized displacement - time history of ankle mobilization / adjustment graphically demonstrating parameters used as KRTF to improve the performance of the technical execution

The experimental protocol of the present study was divided into different phases. The professor, with over 15 years’ experience in manual therapy, performed a detailed explanation about the execution of techniques and different aspects to be taken into account. Next, a demonstration of the technique was carried out for all students and the interpretation of the graph resulting from the kinematic registration was explained. When the explanation/demonstration time was finished, the first parameterized execution for each student of the selected technique was performed. After finishing the first registration, each student practised intensely for 90 min (45 min for each movement, ankle dorsiflexion and ankle plantar flexion) according to the random methodology assigned (G1: traditional method group and G2: KRTF group). The traditional teaching method included an expert professor supervising the students’ practice, the professor-student ratio was 1:8. Students of G2 were placed in front of a station where, while they performed the manoeuvre, they received a KRTF in which the kinematic technique characteristics were observed on a laptop. After 90 min of intense practice, the second parameterized execution of each student in the selected technique was performed. During the parameterized execution of techniques (pre- and post-intervention), each student performed the mobilization 10 times consecutively. Within this cycle of mobilizations, repetitions five, six and seven were measured. Records of kinematic analysis were conducted by a blinded investigator with over 8 years’ experience in kinematic records analysis. A schematic of a graphical KRTF example is shown in Fig. 2.

### Outcome measures

For recorded raw data, the start and end of the mobilization and the maximum range of motion for each of the three axes were identified using an algorithm. Once the three points were located, the following outcome variables were extracted offline: total time of mobilization, time to reach maximum amplitude, maximum angular displacement in the three axes (yaw-pitch-roll), maximum and average velocity to reach the maximum angular displacement, average velocity during the whole execution of the mobilization.

Outcome variables were extracted from the records made by the two IS (leg and heel) for the two mobilizations (ankle dorsiflexion and plantar flexion). Participants’ assignment to the groups, extraction of the variables’ values and data analysis were performed by an independent and blinded researcher.

### Data analysis

A descriptive analysis of the sample was performed with basic anthropometric variables (age, weight, height, body mass index (BMI)) included. An intra-group and inter-group differences analysis was performed pre- and post-intervention. A repeated measures ANOVA was performed for intra-group analysis (pre and post-intervention). The intergroup analysis used Wilcoxon’s test or the Student *t*-test according to the sample distribution (analysed by Kolmogorov-Smirnov test). Student *t*-test was used for variables with a normal distribution (parametric), while for non-parametric variables the Wilcoxon’s test was used. The level of significance was established at *p* ≤ 0.05. A consistency analysis of each outcome variable was performed by considering a test-retest standard deviation of differences (95 % confidence interval (CI)). Measures of consistency were performed with the three pre and post-intervention registers using Cronbach’s alpha (95 % CI). Measures of consistency were stratified into different levels: excellent (Cronbach’s α > 0.80), good (0.80 > Cronbach’s α > 0.60), moderate (0.60 > Cronbach’s α > 0.40) or poor (Cronbach’s α <0.40)[32]. Regarding sample size, a minimum of 54 participants (27 per group) was necessary according to the calculation of sample size (using EPIDAT 3.1 software) with an error rate of 0.5 and a 95 % CI. The Statistical Package for Social Sciences (SPSS V-21) was used to perform the statistical analysis.

## Results

Sixty students (G1: 30 (15 men/15 women) and G2: 30 (15 men/15 women)) participated in the present study. No significant differences between groups in any of the anthropometric variables were found. The average age of the sample was 22.04 (±1.59) years, a weight of 69.59 (±9.27) kg, a height of 171.30 (±12.88) cm and BMI of 23.68 (±4.24) kg/m^2^, respectively.

**Fig. 3:**
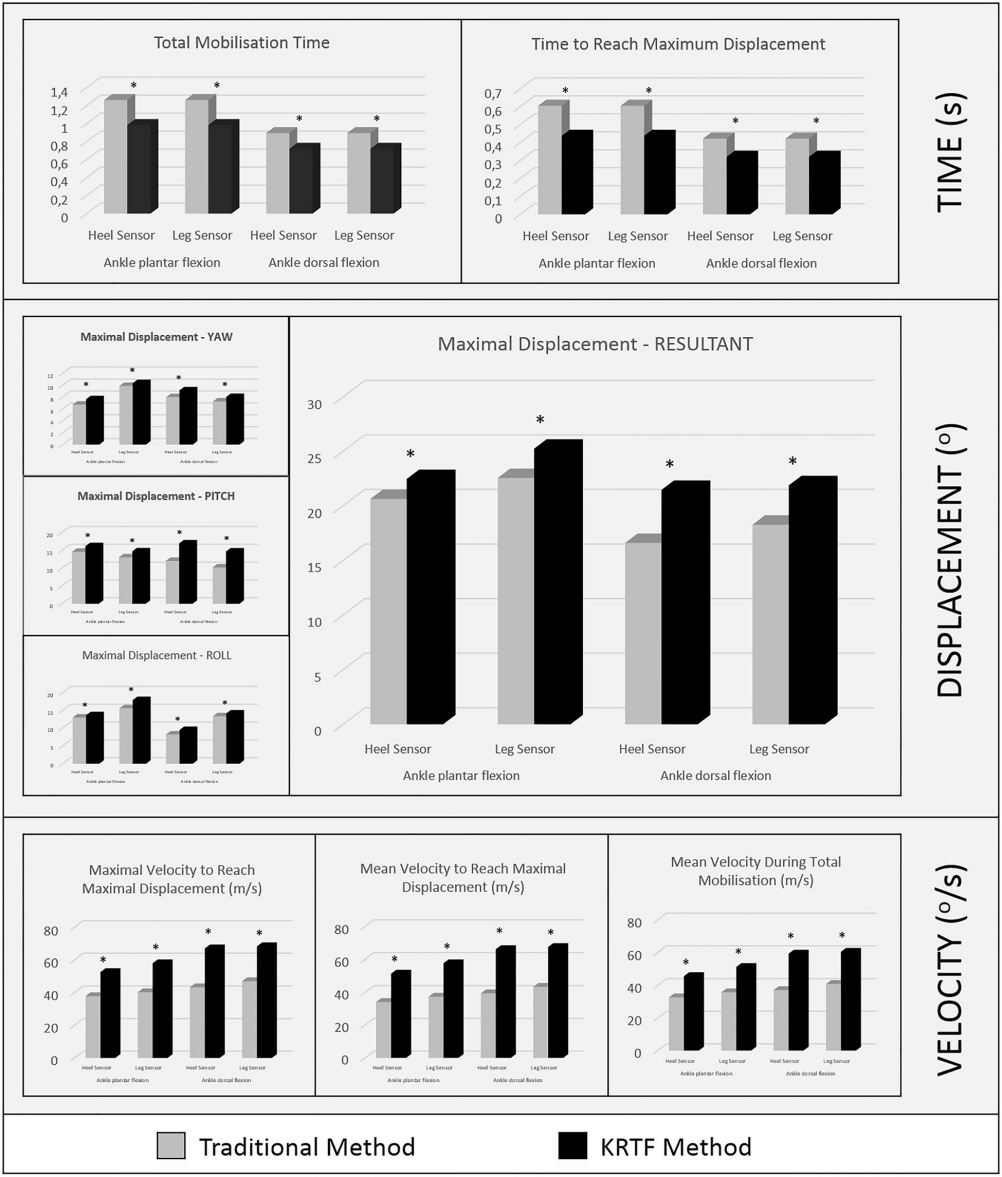
Inter-group kinematic variables comparison recorded post-intervention during ankle plantar flexion and ankle dorsal flexion

Tables 1 and 2 show intragroup differences in kinematic records during ankle plantar flexion. Among the pre-post intervention measurements, there were significant differences within the two groups for all outcome variables. Similarly, the progression made by the two groups after the intervention during dorsal ankle mobilization revealed significant differences between the outcome variables for pre- and post-intervention measures. In intergroup comparisons, there were no significant differences in any of the outcome variables for measurements performed before the intervention. The magnitude of progress made was not similar between groups (Fig. 3). Within the figure, the post-intervention measurements were significantly different for all outcome variables for the experimental group during ankle dorsiflexion, such as ankle plantar flexion.

**Table 1:** Kinematic variables extracted during ankle plantar flexion with the sensor placed in the heel. Results calculated thought an ANOVA with repeated measures

		G_1_ (SD)				G2 (SD)			
		PRE	POST	DIFFER	F	PRE	POST	DIFFER	F
TMT(s)		1.496 (±0.134)	1.262 (±0.155)	-0.234 (±0.033)	17.384***	1.423 (±0.115)	0.984 (±0.106)	-0.439 (±0.040)	79.642***
TRMD(s)		0.690 (±0.059)	0.604 (±0.053)	-0.086 (±0.007)	7.248**	0.704 (±0.063)	0.437 (±0.059)	-0.267 (±0.032)	184.642***
MD(°)	Yaw	8.440 (±0.903)	6.723 (±0.787)	0.451 (±0.038)	3.846**	6.403 (±0.591)	7.643 (±0.803)	1.240 (±0.140)	59.846***
	Pitch	11.379 (±1.204)	14.576 (±1.722)	2.125 (±0.199)	4.268*	12.170 (±1.364)	16.156 (±1.920)	3.986 (±0.420)	84.335***
	Roll	13.334 (±1.409)	12.964 (±1.440)	1.001 (±0.093)	3.887**	11.871 (±1.596)	13.614 (±1.637)	1.743 (±0.190)	79.164***
	Resultant	18.704 (±2.073)	20.633 (±2.359)	1.929 (±0.138)	3.965*	19.060 (±2.035)	22.467 (±2.501)	3.407 (±0.288)	84.125***
VRMD (°/s)	Maximal	35.544 (±3.420)	37.929 (±4.196)	2.385 (±0.204)	8.145**	34.917 (±3.388)	52.698 (±5.571)	17.781 (±1.958)	67.421***
	Mean	27.107 (±2.964)	34.160 (±3.699)	7.053 (±0.624)	6.254*	27.074 (±2.937)	51.411 (±5.270)	24.337 (±2.771)	74.110***
VTM (°/s)	Mean	25.005 (±2.735)	32.638 (±3.607)	7.633 (±0.723)	7.684	26.788 (±2.993)	45.664 (±4.530)	18.876 (±2.034)	33.576***

*MD* Maximum displacement, *TMT* total mobilization time, *TRMD* time to reach maximum peak, *VRMD* velocity to reach maximum displacement, *VTM* Velocity during total mobilization

Signification level:* ≤ 0.05, ** ≤ 0.01, *** ≤ 0.001

**Table 2:** Insert Kinematic variables extracted during ankle plantar flexion with the sensor placed in the leg. Results calculated thought an ANOVA with repeated measures

		G_1_ (SD)				G2 (SD)			
		PRE	POST	DIFFER	F	PRE	POST	DIFFER	F
TMT(s)		1.496 (±0.167)	1.262 (±0.144)	-0.234 (±0.026)	14.698**	1.423 (±0.151)	0.984 (±0.115)	-0.439 (±0.051))	168.431***
TRMD(s)		0.690 (±0.073)	0.604 (±0.068)	-0.086 (±0.009)	16.288**	0.704 (±0.073)	0.437 (±0.049)	-0.267 (±0.029)	206.479***
MD(°)	Yaw	8.440 (±0.903)	9.866 (±1.006)	1.426 (±0.153)	6.374*	8.594 (±0.830)	10.356 (±0.977)	1.762 (±0.173)	96.387***
	Pitch	11.379 (±1.204)	13.025 (±1.401)	1.646 (±0.177)	6.244*	10.969 (±0.996)	14.724 (±1.560)	3.755 (±0.360)	40.365***
	Roll	13.334 (±1.409)	15.544 (±1.611)	2.210 (±0.237)	6.972*	13.128 (±1.429)	17.846 (±1.169)	4.718 (±0.509)	65.799***
	Resultant	19.455 (±2.113)	22.552 (±2.305)	3.097 (±0.319)	7.983**	19.144 (±2.201)	25.248 (±2.721)	6.104 (±0.622)	74.301***
VRMD (°/s)	Maximal	32.048 (±3.331)	40.325 (±4.197)	8.277 (±0.860)	5.379*	30.248 (±3.288)	58.104 (±6.103)	27.856 (±2.987)	69.876***
	Mean	28.196 (±3.001)	37.338 (±3.804)	9.142 (±0.925)	11.348**	27.193 (±2.877)	57.776 (±6.013)	30.583 (±3.122)	74.255***
VTM (°/s)	Mean	26.009 (±2.734)	35.740 (±3.422)	9.731 (±0.986)	12.344**	26.907 (±2.589)	51.317 (±6.579)	24.410 (±2.207)	35.670***

*MD* Maximum displacement, *TMT* total mobilization time, *TRMD* time to reach maximum peak, *VRMD* velocity to reach maximum displacement, *VTM* Velocity during total mobilization

Signification level:* ≤ 0.05, ** ≤ 0.01, *** ≤ 0.001

**Fig. 4:**
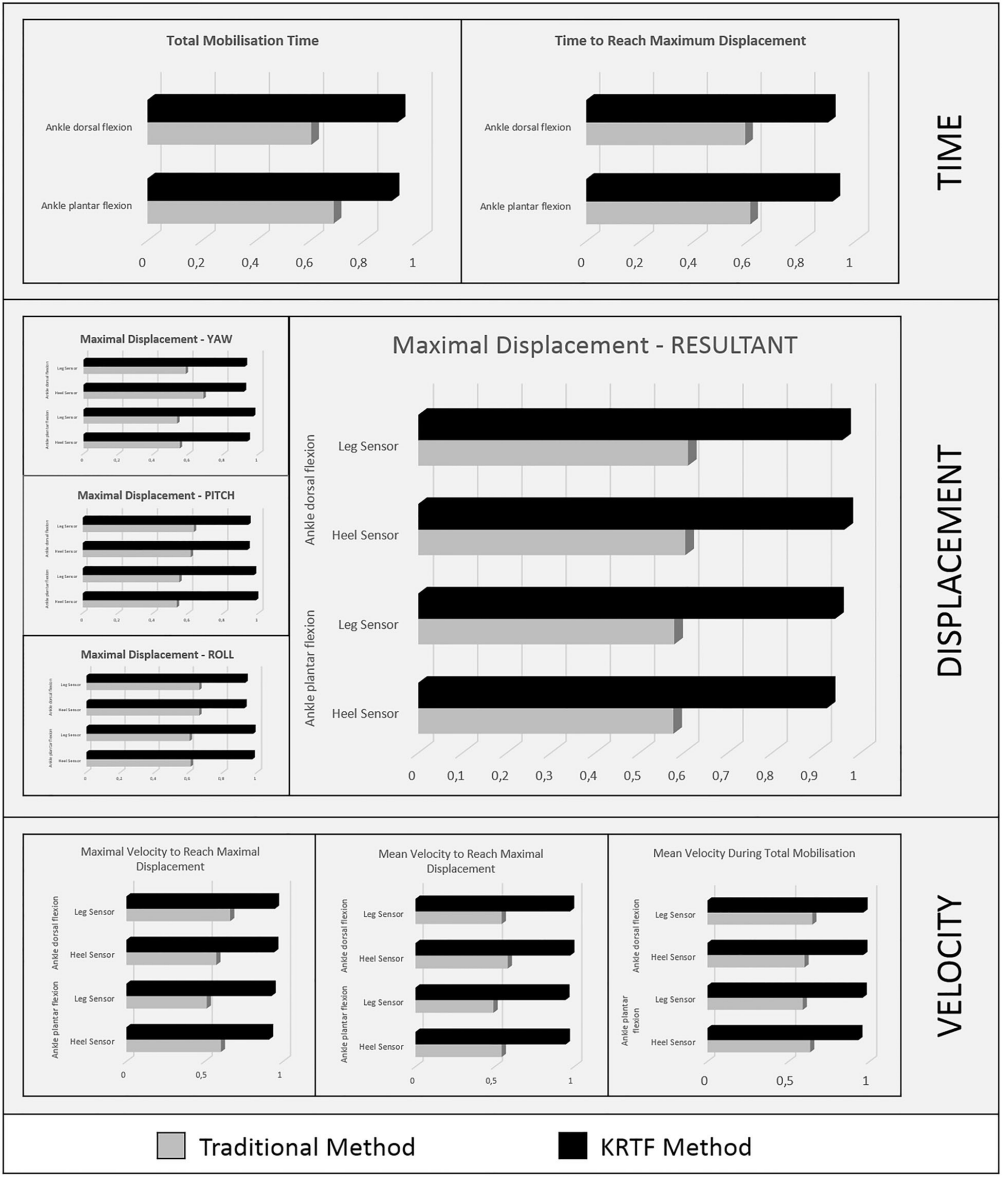
Stability measurement of kinematic variable obtained during plantar and dorsal flexion postintervention

Records conducted post-intervention were much more stable in G2 than in G1 (Fig. 4). Although values (Cronbach’s alpha (95 % CI)) were comparable in the first measurements (pre-intervention) for both sensors on both mobilizations, this was not the case for post-intervention reliability. For mobilization in ankle plantar flexion, the measurement stability of G1 ranged between 0.491 (velocity to reach maximum displacement-leg sensor) and 0.687 (total manipulation time), while the measurement stability of G2 ranged between 0.899 (time to reach maximum displacement) and 0.984 (maximal displacement (pitch)-heel sensor) (Fig. 4). In the same way, measurements taken during ankle dorsal flexion mobilization were similar to G1 where the measurement stability (post-intervention) ranged from 0.543 (velocity to reach maximum displacement-leg sensor) and 0.684 (maximum displacement (yaw)-heel sensor) while G2 ranged between 0.899 (time to reach maximum displacement) and 0.974 (velocity to reach maximum displacement (mean)-heel sensor) (Fig. 4).

## Discussion

The aim of the present study was to analyse the effect of the teaching-learning process with the use of two peripheral joint mobilization techniques (ankle dorsiflexion and plantar flexion with talus displacement), as an example of new manual ability/skill that must to be performed accurately and swiftly, which can potentially benefit from KRTF as a strategy for learning this skill. Comparing the KRTF method with the traditional teaching method, the students who used KRTF were significantly different from students who used the traditional method, as seen in the kinematic variables, such as stability measures. Thanks to the results obtained, the aim of this study was achieved and the hypothesis was confirmed.

While to the best of the authors’ knowledge this is the first study to use real-time feedback in the teaching-learning of peripheral joint mobilizations, the results obtained were consistent with previous studies that used real-time feedback with high-speed and low amplitude techniques on the spine. Both study groups achieved improved kinematic parameters of mobilizations (Tables 1 and 2), however G2 (KRTF) achieved significantly greater improvements in the kinematic parameters for the two mobilizations (significant increase in displacement and velocity, and a significant reduction in the mobilizations’ runtime) than G1 (traditional method) (Fig. 3). Also in the kinematic parameters’ modification of the peripheral joint mobilization, there was an increased stability of the execution in all outcome variables (Fig. 4).

### Consistency of measures

The consistency obtained in both groups in the measurements recorded during pre-intervention mobilizations reached levels between poor and moderate (Fig. 4) [20]. However, after the intervention, the consistency of measures in both groups changed considerably. Thus, for G1 (traditional method), the consistency of measures improved slightly, ranging from moderate to good. On the other hand, consistency values observed in G2 (KRTF) were excellent [20], due to all kinematic variables reaching values of Cronbach’s alpha (95 % CI) greater than 0.901 (total mobilization time) and 0.899 (time to reach maximum displacement) during ankle plantar flexion and ankle dorsiflexion, respectively. A previous study showed consistency levels comparable to those demonstrated by two therapists with over 7 years’ experience in manual therapy [16], with inter-class correlation values between 0.808 and 0.928, although these values were achieved by manipulation of the cervical spine. Measures of consistency are very important because they are evidence that, in the same situation, the therapist is able to always provide the same answer, one of the principal characteristics that distinguish expert manual therapists [4]. Furthermore, this trend to improve the consistency of mobilization execution was congruent with previous studies that showed similar improvements when the student performs the learned practice thanks to real-time feedback, either from IS [5] or an instrumented treatment table [4, 6]. This improvement in measure consistency may be because students, who have an objective information system about the correct execution technique, are able to identify mistakes and correct them, thus increasing their autonomy in the teaching-learning process and the number of useful repetitions during the practice.

### Intra-group and inter-group results

Analysing the results observed after the practice period of ankle plantar flexion and dorsiflexion mobilizations (with talus sliding), and comparing both intervention groups, all outcome variables achieved significant differences between pre-intervention and post-intervention measurements (Tables 1 and 2). However, when analysing inter-group outcome variables, the magnitude of change in execution was significantly higher in G2 (KRTF) than in G1 (traditional methods) (Fig. 3). This same change trend in the results, comparing intra- and inter-group, was in line with a previous published study [5]. In both studies, the instrument generated real-time feedback via an IS, and in both studies the traditional teaching method of manual therapy and the KRTF method were compared, although the manoeuvre chosen was different: two techniques to mobilize the ankle (ankle dorsiflexion and plantar flexion with talus displacement) in the present study, and posterior-anterior thoracic manipulation in the other [5].

### Transferability to other areas in medical education

This study presents the results of an innovative methodology-kinematic real-time feedback (KRTF)-on the teaching and learning of specific mobilization of the ankle. The aim is to optimize the learning period for students in developing new manual skills necessary in the development of their work through an immediate kinematic feedback shown to the student during the mobilization.

This methodology could have great potential and prove easily transferable to other medical areas. For example, there are numerous techniques for assessing and treating neuro-muscular-skeletal structures in different medical areas, such as physical medicine, orthopaedics, dentistry, podiatry and physiotherapy. The development of these techniques could easily be broken down into basic kinematic parameters, for example (linear and/or angular) displacement and time, as well as certain variables derived from these, such as speed and/or acceleration. This decomposition allows kinematic benefits from the principles of the KRTF methodology.

Similarly, in the surgical field, accuracy in both the initial positioning of the hand when making an incision and displacement during execution is critical to the success of the surgical procedure [21–24]. The learning of these techniques, at least in the early stages, could benefit from the transfer of this new methodology as its development can also be decomposed into kinematic parameters. Just as with the making of incisions, the movement of the hand during suturing can also be decomposed into kinematic parameters, so that the transfer of the principles of KRTF could also improve the learning process of this technique. Hence, all medical disciplines related to surgery could benefit from the transfer of this methodology.

Finally, a real-time feedback has proved effective in the teaching and learning of cardio-pulmonary resuscitation [25], a technique that should be familiar to and correctly implemented by all health professionals. Kinematic real-time feedback, could be very useful for teaching/learning this skill, due it should be done with a specific timing and rhythm.

In summary, KRTF is a methodology that could be transferred to the teaching/learning and practice of manual skills in different medical areas, such as general medicine, podiatry, dentistry, physiotherapy, nursing, occupational therapy, etc. As an alternative to the traditional methodologies of learning of these manual skills, KRTF could improve student autonomy in the learning process, as well as improving consistency in the execution of manual techniques, based on the results obtained in this study. Future studies are needed to confirm the hypothesis that the same gains will hold in other manual skills that are part of the responsibilities of the various medical areas.

### Strengths and weaknesses

To the best of the authors’ knowledge this is the first study to use real-time feedback as a teaching strategy for two mobilisations in peripheral joints. In addition, one of the main strengths was the fact that real-time feedback was performed with IS. This feature is important for two main reasons: 1) because peripheral joint mobilization is, for the neuromuscular system, a complex skill because it requires a bimanual coordination to perform an accurate and sudden movement and KRTF helps to improve the results of its learning process. From a biomechanics point of view, these kinds of abilities/skills are usual in all health professions, so the results of the present study could be extrapolated and integrated into the learning process of other health professions, such as medicine, nursing, podiatry, dentistry, etc.; 2) it overcomes one of the main limitations of force-recording devices (stretch manipulation treatment, handheld force transducer or instrumented manikin), performing registration in two dimensions when the forces during a mobilization take place in three dimensions. Another strength of this study is that the use of the KRTF method promoted an increase in the number of repetitions students can perform in an autonomous way during the learning practice. However, the present study also had some weaknesses, such as the immediate effect of the intervention analysed; hence it would be necessary to design studies that observed the skills acquired by examining short-, medium- and long-term retention.

## Conclusion

KRTF could be a useful tool in the teaching-learning process of two joint mobilization techniques (ankle dorsiflexion and plantar flexion with talus displacement towards anterior and posterior, respectively), showing a significant improvement in the ability to perform these manual abilities/skills, which means, in kinematic terms, an increase of joint displacement and execution velocity on the one hand, and on the other hand, a reduction in runtime. To use a customised system in real time could allow the student to analyse the execution of the movement itself, encouraging reflection and self-correction of the manipulation, increasing the number of repetitions and the perception of any faults while performing the manipulation, and thereby favouring increased autonomy of the students during learning.

The principles of this learning-teaching process could be extrapolated and integrated to other manual abilities/skills associated with or relevant to other health professions.

## Abbreviations

BMI: Body mass index
CI: Confidence interval
IS: Inertial sensor
KRTF: Kinematic real-time feedback
